# Function Formula Oriented Construction of Bayesian Inference Nets for Diagnosis of Cardiovascular Disease

**DOI:** 10.1155/2014/376378

**Published:** 2014-08-27

**Authors:** Booma Devi Sekar, Mingchui Dong

**Affiliations:** Department of ECE, Faculty of Science & Technology, University of Macau, Avenue Padre Tomas Pereira S.J.S, Macau

## Abstract

An intelligent cardiovascular disease (CVD) diagnosis system using hemodynamic parameters (HDPs) derived from sphygmogram (SPG) signal is presented to support the emerging patient-centric healthcare models. To replicate clinical approach of diagnosis through a staged decision process, the Bayesian inference nets (BIN) are adapted. New approaches to construct a hierarchical multistage BIN using defined function formulas and a method employing fuzzy logic (FL) technology to quantify inference nodes with dynamic values of statistical parameters are proposed. The suggested methodology is validated by constructing hierarchical Bayesian fuzzy inference nets (HBFIN) to diagnose various heart pathologies from the deduced HDPs. The preliminary diagnostic results show that the proposed methodology has salient validity and effectiveness in the diagnosis of cardiovascular disease.

## 1. Introduction

Cardiovascular diseases (CVD) are known as the silent killers and often they may develop over time without being noticed until a critical stage is reached. Early diagnosis, care, and continuous monitoring are crucial in preventing heart failures. Thus, exploiting the benefit of multiple technological advancements, research over the past decade has focused on the development of various intelligent tools, to support healthcare professionals and promote CVD self-monitoring. In the same vein, our research team has also been devoted to the research and development (R&D) of e-home healthcare system for CVD self-monitoring [1–5]. One of our key developments is the hemodynamic analysis of sphygmogram (SPG) signal [3], which derives 32 critical vital signs/hemodynamic parameters (HDPs). These HDPs such as cardiac output (CO), stroke volume (SV), systematic arterial compliance (SAC), total peripheral resistance (TPR), and so forth can serve as indices to monitor the health status of cardiovascular system [3, 6, 7].

Though the benefits of SPG and hemodynamic analysis have been well documented [8–11], we believe that an intelligent CVD diagnosis system based on the derived HDPs would benefit e-home healthcare. In this paper, we propose to apply artificial intelligence (AI) technology to develop such an intelligent CVD diagnosis system. Literature review shows that among various AI technologies, expert system (ES), in particular Bayesian inference nets (BIN), has emerged as one of the most successful intelligent tools in various applications [12–14]. Especially, BIN with its ability to execute staged decision process and provide reasoned conclusions has established a long track record in medical informatics [15–19], leading to the development of various clinical decision support systems (CDSS) [20–24]. To support a doctor's approach of diagnosis with staged decision process, a BIN is adopted in this paper in order to design an intelligent CVD diagnosis system based on HDPs.

However, difficulty arises in constructing the BIN and quantifying the inference nodes to compute the inference through the nets and solve uncertainties. Many renowned researchers including Pearl [25], Chickering [26], Heckerman [27], Friedman and Koller [28], and so forth have contributed towards addressing such bottleneck problem. Some of the key contributions in this regard are worth mentioning.

In constructing a BIN, researchers first developed algorithms that learn the parameters from a large data set to optimally construct the graphical model. These were generally referred to as the learning models and were further distinguished into search and score based methods [26, 29] and conditional dependence analysis methods [30]. In such methods, although both the graphical topology and the joint probability distribution could be learnt and defined from the data set, there were many shortcomings as implementation of such approach required large amount of qualified data.

The alternative to the data-driven approach was the manual construction of BIN through knowledge acquisition from domain experts using various knowledge elicitation techniques [31]. Though, initially, it was the preferred approach for developing CDSS, it also suffered subsequently from various challenges. It became challenging to systematically analyze the acquired knowledge to construct a hierarchical multistage BIN. Moreover, parameter estimation by different experts faced cognitive biases, often leading to ad hoc estimation of a large number of statistical parameters (e.g., prior probabilities, likelihoods, etc.). Moreover, manual construction required the prior specification of graphical structure between domain variables.

To overcome the critical challenges, some unique benefits from data-driven and knowledge elicitation techniques are availed in this paper and a new approach to construct hierarchical multistage BIN and quantify the inference nodes is proposed. Function formulas in first order predicate logic form are derived to guide in constructing the hierarchical multistage BIN. Further, the FL technology is used to quantify dynamic statistical parameters to inference nodes. The proposed methodology is then applied to construct hierarchical multistage Bayesian fuzzy inference nets (HBFIN) to diagnose various heart pathologies based on HDPs. HBFIN is finally validated using site-measured medical data acquired from two hospitals in China.

## 2. Hemodynamic Parameters

**Figure 1:**
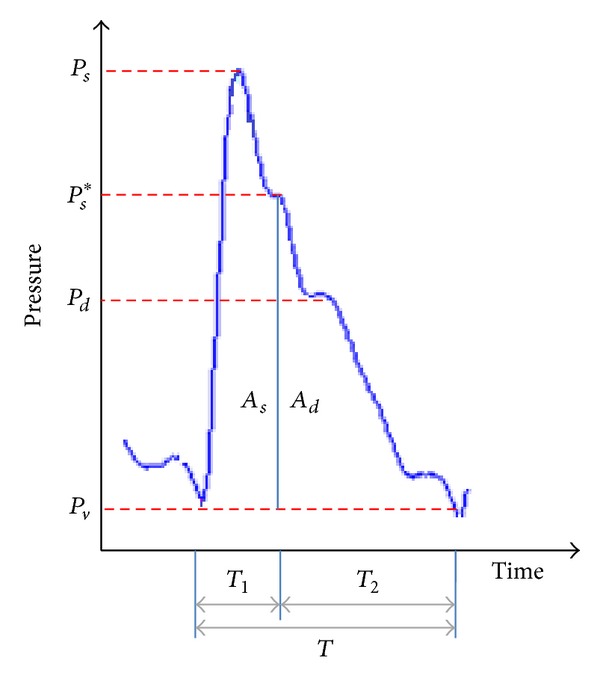
Point and area based morphological features of a typical SPG signal.

HDPs derived from hemodynamic analysis of SPG signal can serve as powerful indices for prognosis of CVDs. There are various approaches to hemodynamic analysis [32, 33]. In this paper, hemodynamic analysis is computed based on elastic cavity theory [3], in which the point and area based morphological features of SPG signal as shown in Figure 1 are used to deduce 32 HDPs. The following will show the derivation of some of the important HDPs.

Blood flow continuous equation is

(1) $$\begin{array}{c} Q_{\text{in}}=Q_{\text{out}}+\frac{dV}{dt_{1}}, \end{array}$$

(2) $$\begin{array}{c} Q_{\text{out}}+\frac{dV}{dt_{2}}=0, \end{array}$$

where *Q*_in_ is the volume of blood flowing into the artery and *Q*_out_ is the volume of blood flowing into the vein. *t*_1_ and *t*_2_ are the systolic and diastolic time period, respectively.

Relation between pressure and blood flow is

(3) $$\begin{array}{c} Q_{\text{out}}=\frac{p-p_{v}}{R}, \end{array}$$

where *p* is the arterial pressure, *p*_*v*_ is the venous pressure, and *R* is the peripheral resistance of cardiovascular system.

Arterial pressure-volume equation is

(4) $$\begin{array}{c} \mathrm{AC}=\frac{dV}{dp}, \end{array}$$

where AC⁡ is the arterial compliance.

Now, with (1)~(4), the analytic equation of elastic cavity can be computed as follows:

(5) $$\begin{array}{c} Q_{\text{in}}=\mathrm{AC}\frac{dp}{dt_{1}}+\frac{p-p_{v}}{R}, \end{array}$$

(6) $$\begin{array}{c} \mathrm{AC}\frac{dp}{dt_{2}}+\frac{p-p_{v}}{R}=0. \end{array}$$

Computing the integral of (5) and (6),

(7) $$\begin{array}{c} \text{SV}=\mathrm{AC}\left(P_{s}^{*}-P_{d}\right)+\frac{A_{s}}{R},\\ \mathrm{AC}\left(P_{d}-P_{s}^{*}\right)+\frac{A_{d}}{R}=0, \end{array}$$

where SV is the stroke volume and the parameters *A*_*s*_,*  A*_*d*_,*  P*_s_*, and *P*_*d*_ are the morphological features obtained from SPG as in Figure 1. Thus, the HDPs-SV, AC⁡, *R*, and so forth can be computed with following equations.

Auxiliary blood pressure index is

(8) $$\begin{array}{c} k=\frac{\int_{0}^{T}\bar{P}dt}{T\left(P_{s}-P_{d}\right)}=\frac{\bar{P}-P_{d}}{P_{s}-P_{d}}. \end{array}$$

Stroke volume is

(9) $$\begin{array}{c} \text{SV}=\frac{0.28}{k^{2}}T\left(P_{s}-P_{d}\right). \end{array}$$

Auxiliary sphygmogram index is

(10) $$\begin{array}{c} \eta =\frac{A_{s}+A_{d}}{A_{d}}=1+\frac{A_{d}}{A_{d}}. \end{array}$$

Arterial compliance is

(11) $$\begin{array}{c} \mathrm{AC}=\frac{\text{SV}}{\eta \left(P_{s}-P_{d}\right)}. \end{array}$$

Peripheral resistance is

(12) $$\begin{array}{c} R=\frac{\bar{P}-P_{v}}{\text{SV}}\cdot T\approx \frac{\bar{P}}{\text{SV}}\cdot T. \end{array}$$

Similarly, with morphological features and deduced HDPs, various other HDPs can be generated.

## 3. Define Function Formulas and Statistical Parameters

### 3.1. Medical Data Acquisition

The site-measured medical data consists of medical records of different samples, including each patient's physiological attributes, original SPG waveforms, HDPs, and doctor's clinical diagnostic results. Here, the medical symptom space is denoted by MSS ∈*R* ^*N*^, where *N* = 38, including 6 physiological attributes and 32 HDPs. Totally, 2267 medical records of 165 patients were acquired from two hospitals in China and 850 healthy records were randomly collected. Patient's SPG waveforms and HDPs were measured 12 to 15 times at different time interval within 5 weeks, and their physiological attributes such as age, gender, height, weight, and so forth were also recorded.

Moreover, a medical knowledge base was developed by acquiring information from various medical sources to analyze the relation between the derived HDPs and various pathological conditions of heart. Such medical knowledge base was then verified by doctors from two hospitals in China.

### 3.2. Define Function Formula in First Order Predicate Logic Form

The key step in constructing inference nets is to define the function formulas in first order predicate logic form using the developed medical knowledge base.

Following equation shows an example of such defined function formula:

(13) ∀p·(SPa(p)∧MDPa(p)∧MAPa(p)∧DPa(p)   ⟶HT(p)),

where *p* represents patient. SP, MDP, MAP, and DP are the symptoms and HT is the diagnosed hypothesis. Expansion of acronym/abbreviation of the HDPs and CVDs used in function formulas are presented in Figure 3. The suffix “*a*” represents the value of individual specific condition. The condition values of symptoms for indicating different pathological condition of heart are presented in Table 1.

Deriving from the medical knowledge base, the first order predicate logic formulas for diagnosing various other heart pathologies can be defined as follows:

(14) ∀p·(BVa(p)⟶Low_BV(p)),∀p·(MAPb(p)∧SPb(p)∧Low_BV(p)⟶HPT(p)),∀p·(PRa(p)⟶TC(p)),∀p·(PRb(p)⟶BC(p)),∀p·(SVa(p)∧SIa(p)⟶Low_BE(p)),∀p·(SVb(p)∧SIb(p)⟶High_BE(p)),∀p·(VPEa(p)∧CIa(p)⟶Low_CPP(p)),∀p·(VPEb(p)⟶High_CPP(p)),∀p·(Ya(p)∧Yra(p)⟶HV(p)),∀p·(Yb(p)∧Yrb(p)⟶HPV(p)),∀p·(AC⁡a(p)∧FEKa(p)∧Wta(p)∧BLKa(p)∧SVb(p)∧HV(p)⟶Dyn_HV(p)).

The defined function formulas can then be used to guide in constructing the hierarchical multistage inference nets to diagnose various CVDs.

### 3.3. Quantification of Inference Nodes with Dynamic Statistical Parameters

Based on the data distribution, various types of function such as Gaussian, triangle, high-order polynomial, *S*-type, and so forth can be employed to define the FL membership functions (MF). In the proposed approach, based on the histogram obtained from the frequency plot of observed medical records, high-order polynomial, *S*-type, or quasi-Gaussian functions are adapted to define the individual MF.

The general formula of *i*th MF *f*_*i*_(*s*_*j*_^(0)^) versus *j*th symptom in 0th stage *s*_*j*_^(0)^ expressed in high-order polynomial, *S*-type, or quasi-Gaussian functions are sequentially listed below:

(15) $$\begin{array}{c} f_{i}\left(s_{j}^{\left(0\right)}\right)=\lambda_{0}+\lambda_{1}s_{j}^{\left(0\right)}+\lambda_{2}{\left(s_{j}^{\left(0\right)}\right)}^{2}+\cdots +\lambda_{t}{\left(s_{j}^{\left(0\right)}\right)}^{t}, \end{array}$$

where *t* is the order of polynomial, *λ*_0_ is random error or noise component, and *λ*_1_, *λ*_2_,…, *λ*_*t*_ are coefficients. Consider

(16) $$\begin{array}{c} f_{i}\left(s_{j}^{\left(0\right)}\right)=\frac{1}{1+e^{-a(s_{j}^{\left(0\right)}-b)}}, \end{array}$$

where *b* is the turning point of curve and *a* is the slope of function. Consider

(17) $$\begin{array}{c} f_{i}\left(s_{j}^{\left(0\right)}\right)={e^{-(\left(s_{j}^{\left(0\right)}-a_{i}^{}(s_{j}^{\left(0\right)})\right)/2d_{i}^{}(s_{j}^{\left(0\right)}))}}^{2}, \end{array}$$

where *a*_*i*_(*s*_*j*_^(0)^) is the maximum membership grade and 2*d*_*i*_(*s*_*j*_^(0)^) is the bandwidth of that function. Here, the word “quasi” is expressed to indicate that the MF plot would appear as Gaussian distribution shape, but will not cover to the extent of positive and negative infinity.

**Figure 2:**
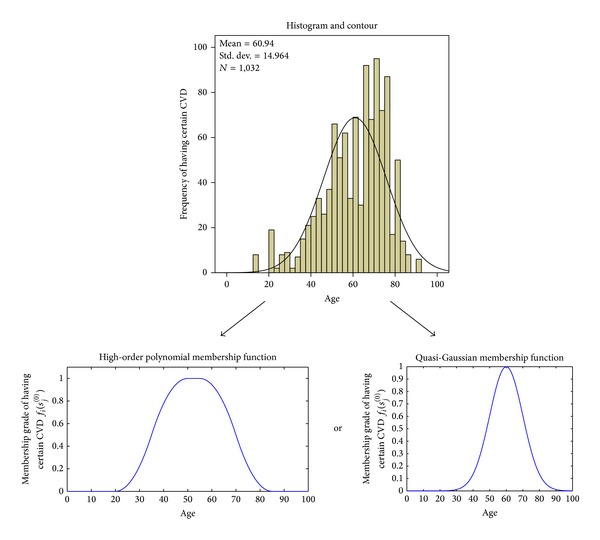
Generation of high-order polynomial or quasi-Gaussian membership function for symptom* Age* versus certain CVD.

Based on statistical analysis of site-measured records, the MF for each plot of pair (symptom (HDP) versus membership grade of having specific CVD) is predefined. This therefore fixes all the parameters of (15), (16), or (17) for that particular MF. With an example, Figure 2 illustrates how the MF can be defined from the statistical analysis of site-measured records. Thus, by using such predefined MF, whenever a new patient is tested in the constructed BIN, the statistical parameters can be automatically computed and assigned to relevant symptom node in the inference nets using (15), (16), or (17). It is noteworthy that in this paper a hold-out validation was adopted, whereby for each CVD condition 75% of the acquired data set is used for defining the MF, and the remaining samples are used for validating the constructed BIN.

Now, in HBFIN, when testing data *s*_*j*_^(0)^ is recorded, it will be substituted in (15), (16), or (17) to compute the relevant *f*_*i*_(*s*_*j*_^(0)^). This membership grade dynamically varies according to the value of each symptom, and it approximately reflects the effect of that symptom in diagnosing the CVD. With *f*_*i*_(*s*_*j*_^(0)^), dynamic values of statistical parameters are then defined by following rules 1~2 and assigned to the symptom node automatically.

*Rule 1*. IF *f*_*i*_(*s*_*j*_^(0)^) ≥ 0.2, then *P*_*i*_(*s*_*j*_^(0)^) = *f*_*i*_(*s*_*j*_^(0)^) and LS_*i*_(*s*_*j*_^(0)^) = *α*∗*f*_*i*_(*s*_*j*_^(0)^). 

*Rule 2*. IF *f*_*i*_(*s*_*j*_^(0)^) < 0.2, then *P*_*i*_(*s*_*j*_^(0)^) = *f*_*i*_(*s*_*j*_^(0)^) and LN_*i*_(*s*_*j*_^(0)^) = *β*∗*f*_*i*_(*s*_*j*_^(0)^).

The coefficients *α* and *β*, in rules 1~2, can be experimentally assigned as zero or positive integer values. While a bigger *α* would increase the probability of the hypothesis to be true in presence of the evidence, a smaller *β* would increase the probability of the hypothesis to be false in absence of the evidence. In this paper, the values *α* = 100 and *β* = 10 are experimentally chosen. Also, in order to avoid outliers, a threshold of 0.2 is chosen. Then, the statistical parameters for the intermediate hypothesis nodes are defined and assigned using the principle of indifference (PoI) [34], which states that “*each member of a set of propositions could be assigned the same probability of truth in the absence of any reason to assign them different probabilities*.”

## 4. Hierarchical Bayesian Fuzzy Inference Nets to Diagnose Cardiovascular Diseases

### 4.1. Construction of Hierarchical Bayesian Fuzzy Inference Nets

**Figure 3:**
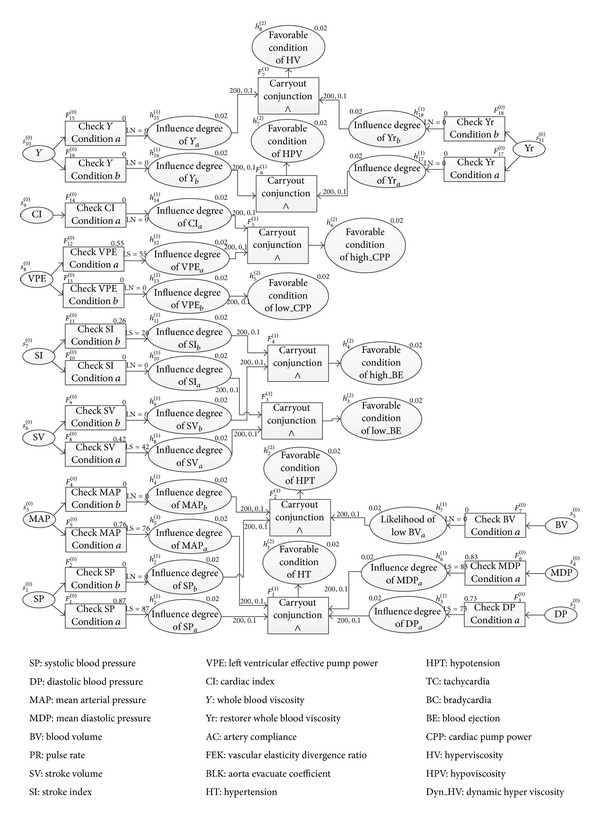
Partially constructed HBFIN for diagnosing heart pathologies with statistical parameters assigned for a sampled medical record.

With the function formulas defined in (13)–(14), the symptoms and intermediate and final hypotheses nodes can be identified. Subsequently, the inference nodes can be generated step-by-step and appropriately linked to construct hierarchical multistage inference nets. Figure 3 shows the partially constructed HBFIN to diagnose heart pathologies based on HDPs. In order to clearly illustrate the construction of BIN, the inference nets for healthy condition have not been presented in Figure 3.

It is worth emphasizing here that the partially constructed HBFIN in Figure 3 can be further developed for diagnosing various CVDs.

### 4.2. Quantifying Inference Nodes of HBFIN with Statistical Parameters

Generally, the inference nodes are quantified by static values of statistical parameters using subjective (experts' estimation) approach. However, since confliction exists among experts' opinions, defining appropriate static values of statistical parameters to inference nodes has always been a challenge. But, with the proposed methodology using FL technology, dynamic values of statistical parameters can be defined and assigned to inference nodes automatically.

Here, with a specific example, by testing a patient's medical record (partially shown in Table 2) in HBFIN to diagnose CVD, we demonstrate how dynamic values of statistical parameters can be defined/assigned automatically. When the testing data is presented into the symptom nodes of HBFIN, it will be automatically substituted in predefined (15), (16), or (17) to calculate the relevant membership grade *f*_*i*_(*s*_*j*_^(0)^), which will then be used in rules 1~2 to define/assign statistical parameters. Figure 3 shows the partially constructed HBFIN with statistical parameters assigned for the sampled testing data shown in Table 2. It is important to note that, with this approach, the statistical parameters assigned to symptom nodes would dynamically change according to the variation of symptoms. The statistical parameters for the intermediate hypothesis or conclusion nodes are defined and assigned using the PoI. As a result, it can be noted that the intermediate hypothesis nodes are assigned with a prior probability = 0.02, LS = 200, and LN = 0.01, respectively, in HBFIN. For inference nodes executing conjunction and disjunction operations, the statistical parameters would be later calculated from the evidences contributing to these nodes according to Bayesian theory.

## 5. Evaluation of Constructed Bayesian Fuzzy Inference Nets

### 5.1. Mathematical Inference Model Using Bayesian Theory

The Bayesian inference nets generally form a static knowledge structure, in which the probability associated with each inference node consequently changes when the evidence is certain or uncertain. This change in probability is propagated up stage by stage through the hierarchical Bayesian inference nets to ultimately support or disprove the top-level hypothesis/conclusion. In this paper, the following inference model is used to compute the inference through the nets. In this model, for addressing uncertainty in evidence, conditional independence of the evidence is assumed. Therefore, for partially known or uncertain evidence, according to its degree of belief, it is categorized as true or false and the inference through the nets is computed accordingly.

(1) Prior odds of *k*th hypothesis on *q*th stage are
  (18) $$\begin{array}{c} O\left(h_{k}^{\left(q\right)}\right)=\frac{P\left(h_{k}^{\left(q\right)}\right)}{1-P\left(h_{k}^{\left(q\right)}\right)}. \end{array}$$
 
*P*(*h*_*k*_^(*q*)^) = prior probability.
(2) Posterior odds of *k*th hypothesis on *q*th stage are
  (19) $$\begin{array}{c} O\left(h_{k}^{\left(q\right)}\;|\;x\left(\text{or}\right)e\right)=\left[\overset{N}{\underset{i=1}{\prod }}{\text{L}}_{i}\right]O\left(h_{k}^{\left(q\right)}\right). \end{array}$$
 
*x*: evidence is certain;
 
*e*: evidence is uncertain;
 L_*i*_: LS_*i*_ {likelihood of sufficiency}
 L_*i*_: LN_*i*_ {likelihood of necessity}
 L_*i*_: 1 {evidence is unknown}.

(3) Posterior probability of *k*th hypothesis on *q*th stage when the evidence is certain is
  (20) $$\begin{array}{c} P\left(h_{k}^{\left(q\right)}\;|\;x\right)=\frac{O\left(h_{k}^{\left(q\right)}\;|\;x\right)}{1+O\left(h_{k}^{\left(q\right)}\;|\;x\right)}. \end{array}$$
(4) Posterior probability of *k*th hypothesis on *q*th stage when the evidence is uncertain is as follows:
 If 0 ≤ *P*(*x*∣*e*) < *P*(*x*), then
  (21) P(hk(q) ∣ e) =P(hk(q) ∣ x′)+P(hk(q))−P(hk(q) ∣ x′)P(x)P(x ∣ e);
 If *P*(*x*) ≤ *P*(*x*∣*e*) ≤ 1, then
  (22) P(hk(q) ∣ e) =P(hk(q))+P(hk(q) ∣ x)−P(hk(q))1−P(x)[P(x ∣ e)−P(x)].
 
*P*(*x*∣*e*) = probability when evidence is uncertain;
 
*x*′ = evidence is certain to be false.

For conjunction inference node, *P*(*x*) = min⁡[*P*(*S*_1_^(0)^),…, (*S*_*n*_^(0)^), *P*(*h*_1_^(*q*−*t*)^),…, *P*(*h*_*d*_^(*q*−*t*)^), *P*(*F*_*k*_^(*q*−*t*)^)].

For disjunction inference node, *P*(*x*) = max⁡[*P*(*S*_1_^(0)^),…, (*S*_*n*_^(0)^), *P*(*h*_1_^(*q*−*t*)^),…, *P*(*h*_*d*_^(*q*−*t*)^), *P*(*F*_*k*_^(*q*−*t*)^)].

### 5.2. Testing Results

The function and validity of partially constructed HBFIN are examined using the reserved testing samples. The number of samples used for testing and the obtained diagnostic accuracy are presented in Table 3.

It is noteworthy that the partially constructed HBFIN in Figure 3 was further constructed using function formulas to diagnose various CVDs, such as coronary heart disease (CHD), arrhythmia (AR), pulmonary heart disease (PHD), cerebral infarction (CIN), hyperlipemia (HL), and so forth. Thus, in such inference nets, the hypothesis nodes Low_BE, High_BE, Low_CPP, High_CPP, and so forth became the intermediate hypothesis nodes, which were further linked with other symptoms or hypothesis nodes to diagnose various CVDs. The diagnostic accuracy of the complete HBFIN is presented in Table 4.

Considering that the diagnosis results are derived only from the HDPs and physiologic parameters in the proposed noninvasive approach, the above diagnostic accuracy is highly acceptable and therefore is suitable for ehome healthcare usage.

Furthermore, Table 5 shows the comparison results of intelligent CVD diagnosis systems using adapted versions of AI technologies, including neural networks (NN) [35], fuzzy neural networks (FNN) [36], and the proposed HBFIN. For performing a fair comparison, these methods were developed and validated by our research team with the same medical database used in this paper.

The diagnostic results in Table 5 demonstrate that the proposed HBFIN provides comparable performance* viz-a-viz* other intelligent CVD diagnostic systems. Moreover, it is important to emphasize here that HBFIN could distinctly trace back from the final hypothesis to its initial symptoms and provide reasoned conclusion to the user. Whereas, in using the systems based on NN that are black box in nature, such a feedback cannot be provided.

## 6. Conclusion

An intelligent CVD diagnosis system based on HDPs derived from SPG signal is presented in this paper. By availing the benefit of some unique features of hybrid AI, BIN, and FL technologies, an intelligent CVD diagnosis system is proposed. A new approach for constructing hierarchical multistage BIN guided by function formulas defined in first order predicate logic form is proposed. A mathematical inference model using Bayesian theory is presented, and a method using FL technology to quantify dynamic values of statistical parameters to inference nodes is suggested. With the proposed methodology, HBFIN is constructed to diagnose various CVDs based on HDPs. The site-measured medical records from two hospitals of China have been used to design and validate the proposed HBFIN. For such a noninvasive diagnostic approach, the testing results with acceptable diagnostic accuracy in diagnosing six important CVDs prove the suitability of HBFIN for home healthcare usage.

## Figures and Tables

**Table 1:** Condition values of symptoms (HDPs) for indicating pathological condition of heart.

Symptoms (units)	Conditions		
	*a*	*b*	*c*
SP (mmHg)	≥160	<90	=110~130
DP (mmHg)	≥95		=80~90
MAP (mmHg)	>115	<65	=70~100
MDP (mmHg)	>105		=66~96
BV (L)	≤{0.75 ∗ Wt ∗ 0.075}		={0.75 ∗ Wt ∗ 0.075}~{1.25 ∗ Wt ∗ 0.075}
PR (mmHg)	≥104	<50	=60~100
Wt (kg)	>20		=50~80
SV (mL/stroke)	≤{0.8 ∗ (1 + *Q*) ∗ 20 ∗ Q}	≥{1.3 ∗ 1.2 ∗ (1 + *Q*) ∗ 20 ∗ *Q*}	≈{(1 +*Q*) ∗ 20 ∗ *Q*}
SI (mL/stroke/m^2^)	≤0.8 ∗ (1 + *Q*) ∗ 20	≥{1.3 ∗ 1.2 ∗ (1 + *Q*) ∗ 20}	≈(1 + *Q*) ∗ 20
VPE (kg/stroke)	≤{0.8 ∗ 2 ∗ (Wt + 45) ∗ 0.0112}	≥{1.2 ∗ 2 ∗ (Wt + 45) ∗ 0.0112}	≈(2 ∗ Wt + 45) ∗ 0.0112
CI (mL/stroke/m^2^)	≥2.2		={(1 + *Q*) ∗ 1.2}~{(1 + *Q*) ∗ 2}
*Y* (mpa*·*s)	≥{1.1 ∗ 4}	≤{0.85 ∗ 3}	=3~4
Yr (mpa*·*s)	≥{1.1 ∗ 4}	≤{0.85 ∗ 3}	=3~4
AC (*µ*m/mmHg)	≥1.2		≥1.2
FEK	≥{0.9 ∗ 0.25}		=0.35~0.55
BLK	<{0.85 ∗ 0.22}		=0.22~0.26

*Wt: patient's weight in kg.

*Q: 0.0061 ∗  *L* (cm) + 0.0128 ∗ Wt (kg) − 0.1592.

**Table 2:** A patient's partial medical record.

Symptoms (units)	Patient's partial medical record
SP (mmHg)	168
DP (mmHg)	100
MAP (mmHg)	130.98
MDP (mmHg)	113.09
BV (L)	3.5212
PR (mmHg)	68
Wt (kg)	49
SV (mL/stroke)	63.81
SI (mL/stroke/m^2^)	45.54
VPE (kg/stroke)	2.18
CI (mL/stroke/m^2^)	2.7
*Y* (mpa*·*s)	3
Yr (mpa*·*s)	3.8
AC (*µ*m/mmHg)	0.66
FEK	0.11
BLK	0.197

∗The expansion of symptom acronym is provided in Figure 3.

**Table 3:** Diagnostic results of partially constructed HBFIN.

Person's health status	Number of samples	Diagnostic accuracy (%)
HT	53	78
HPT	17	82
Low_BE	13	76
High_BE	17	82
Low_CPP	10	80
High_CPP	18	83
HPV	8	87
HV	13	84

∗The expansion of symptom acronym is provided in Figure 3.

**Table 4:** Overall diagnostic accuracy of HBFIN in CVD detection.

Person's health status	Diagnostic accuracy (%)
Healthy	91
HT	78
CHD	68
AR	73
PHD	65
CIN	72
HL	73
Mixed CVD	58

**Table 5:** Diagnostic accuracy of intelligent CVD diagnosis systems using adapted versions of AI technology.

CVD type	Adapted version of AI technology		
	NN	FNN	HBFIN
CHD	78	65	**68**
HT	70	67	**78**
HL	64	77	**73**
Mixed CVD	—	40	**58**
